# Bronchitis and Its Associated Risk Factors in First Nations Children

**DOI:** 10.3390/children4120103

**Published:** 2017-11-24

**Authors:** Chandima P. Karunanayake, Donna C. Rennie, Vivian R. Ramsden, Mark Fenton, Shelley Kirychuk, Joshua A. Lawson, Raina Henderson, Laurie Jimmy, Jeremy Seeseequasis, Sylvia Abonyi, James A. Dosman, Punam Pahwa, The First Nations Lung Health Project Research Team

**Affiliations:** 1 Canadian Centre for Health and Safety in Agriculture, University of Saskatchewan, 104 Clinic Place, Saskatoon, SK S7N 2Z4, Canada; shelley.kirychuk@usask.ca (S.K.); jal226@mail.usask.ca (J.A.L.); james.dosman@usask.ca (J.A.D.); pup165@mail.usask.ca (P.P.); firstnations.lung@usask.ca (F.N.L.H.P.R.T.); 2 College of Nursing, University of Saskatchewan, 104 Clinic Place, Saskatoon, SK S7N 2Z4, Canada; donna.rennie@usask.ca; 3 Department of Academic Family Medicine, University of Saskatchewan, West Winds Primary Health Centre, 3311 Fairlight Drive, Saskatoon, SK S7M 3Y5, Canada; viv.ramsden@usask.ca; 4 Department of Medicine, University of Saskatchewan, Royal University Hospital, 103 Hospital Drive, Saskatoon, SK S7N 0W8, Canada; mef132@mail.usask.ca; 5 Community A, P.O. Box 250, Montreal Lake, SK S0J 1J0, Canada; rkh538@mail.usask.ca; 6 Community B, P.O. Box 96, Duck Lake, SK S0K1J0, Canada; ljimmy@willowcreehealth.com (L.J.); JSeeseequasis@beardysband.com (J.S.); 7 Department of Community Health & Epidemiology, College of Medicine, University of Saskatchewan, 107 Wiggins Road, Saskatoon, SK S7N 5E5, Canada; sya277@mail.usask.ca

**Keywords:** bronchitis, First Nations, children, parental smoking, mold, dampness

## Abstract

Respiratory diseases, such as bronchitis and pneumonia, are common in First Nations children in Canada. The objectives are to determine prevalence and associated risk factors of bronchitis in children 6–17 years old residing in two reserve communities. The cross-sectional study was conducted in 2013 and children from two First Nations reserve communities participated. The outcome was ever presence/absence of bronchitis. Logistic regression analysis was conducted to examine the relationship between bronchitis and the individual and environmental factors. A total of 351 First Nations children participated in the study. The prevalence of bronchitis was 17.9%. While 86.6% had at least one parent who smoked, smoking inside home was 43.9%. Signs of mold and mildew in homes were high. Prevalence of houses with any damage caused by dampness was 42.2%, with 44.2% of homes showing signs of mold or mildew. Significant predictors of increased risk of bronchitis were: being obese; having respiratory allergies; exposed to parental cigarette smoking; and signs of mold and mildew in the home. There are several modifiable risk factors that should be considered when examining preventive interventions for bronchitis including obesity, smoking exposure, and home mold or dampness.

## 1. Introduction

Respiratory diseases including bronchitis and pneumonia are common in First Nations children in Canada [1]. Prevalence rates of respiratory diseases in First Nations children are higher than in the general population of Canadian children [1]. The prevalence of ever bronchitis for First Nations children in Canada is not well known. An earlier study by Senthilselvan et al. [2] reported that physician-diagnosed ever bronchitis prevalence in children between 5 and 14 years old among Registered Indians was 15.6% in 1998. The authors also observed that prevalence rates of ever bronchitis were higher among Registered Indian children compared to urban or rural non-Aboriginal children during the period of 1991 to 1998 [2]. While results from the First Nations Regional Health Survey (RHS) suggest that approximately 1% of First Nations children in 2008–2010 were diagnosed with ever chronic bronchitis compared to 3.6% in 2002–2003 [3].

Bronchitis is an inflammation of the bronchial tubes, causing excessive swelling and mucus production [4]. Cough, increased expectoration of sputum and shortness of breath are the main symptoms of bronchitis [4]. Bronchitis can be either acute or chronic. Acute bronchitis is caused by the same infection that causes the common cold or influenza and lasts about few weeks [5,6]. Chronic bronchitis is defined as a cough that occurs every day with sputum production that lasts for at least 3 months 2 years in a row [7]. 

Second-hand environmental tobacco smoke (ETS), malnutrition, overcrowding, reduced ventilation, lack of fresh running water, wood smoke, and obesity are known risk factors for bronchitis [1]. These factors have a higher prevalence in First Nations populations [1,3].

One issue for First Nations populations is the indoor environment. Studies have reported that about 30–35% of housing on First Nations reserves in Canada are in need of major repairs [8,9]. Poorly constructed or maintained housing can lead to the collection of moisture, resulting in areas of dampness that are likely to be contaminated with mold [8]. Indoor mold has been associated with allergies and also increases the risk of wheezing in young children [8]. Positive associations between exposure to indoor mold and bronchitis have been reported by several authors [10,11,12]. Both personal and passive cigarette smoking leads to respiratory diseases in First Nations children and adults [13,14,15]. 

Living conditions in many First Nations communities are unacceptable [8] compared to the general Canadian population with respect to housing, well-being, health status, income and wealth, and environmental quality. These conditions are characterized by personal, social and physical factors that can be associated with adverse respiratory outcomes [16,17]. Knowledge about bronchitis in First Nations children living on reserve is limited. In this paper, reveal the prevalence and associated risk factors of the respiratory illness of ever bronchitis (acute or chronic) in First Nations children 6–17 years old residing in two reserve communities. 

## 2. Materials and Methods

### 2.1. Population and Study Design

The study population is First Nations children living in two Saskatchewan reserve communities. First Nations comprise one of the three groups of Aboriginal peoples who are the descendants of the original inhabitants of North America (the other two being Métis and Inuit). Individual First Nations have unique heritages, languages, cultural practices and spiritual beliefs [18,19]. The Government of Canada set apart areas of land called “reserves” for the use and benefit of First Nations people. There are treaties between the Government of Canada and First Nations people to officially set out promises, obligations and benefits for both parties [18].

For this analysis, data from the child component of the baseline survey of the First Nations Lung Health Project (FNLHP) [20] was used. The description of the overall study design related to the child component has been published elsewhere [21] and is consistent with Canadian research ethics guidelines captured in Tri-Council Policy Statement: Ethical Conduct for Research Involving Humans (TCPS2) [22]. Briefly, First Nations elders have guided us since the inception of the FNLHP and are fully aware and supportive of our activities. In all stages of the study, community feedback was considered to resolve the issues that arose. Our project manager had discussions with Elders from both communities regarding the letters of consent, assent and the questionnaire. Face to face meetings (7 December 2012; 18 January 2013; and 7 February 2013) at the schools resulted in verbal support from all the schools. The communities provided input for the development of the children questionnaires. 

Children in kindergarten to Grade 12 from four schools in Community A (1 school) and Community B (3 schools) were eligible to participate in this study. In these four schools, permission to conduct the study was granted by different levels of authorities, including the superintendent of the school divisions, the directors of education, and the school principals. The data collection for the children’s study occurred in 2013.

A survey questionnaire was distributed by classroom teachers to prospective participants’ parents or caregivers. The child study questionnaire included items describing socio-demographics, health status of the child, childhood diseases and other illnesses, lifestyle, home environment, health risk behaviors, access to health care and family history of respiratory health. Study packages were retrieved from the schools by the research nurses two weeks after circulation. When the completed survey was returned, parents and/or caregivers received a CAN$5.00 gift card. Of 603 study packages distributed to children 6–17 years of age, 363 (60.2%) were returned, and of these, 351 (58.2%) included completed surveys. The study protocols were submitted and approved by the University of Saskatchewan’s Biomedical Research Ethics Board (#Bio: 13-27) prior to beginning fieldwork in 2013. 

### 2.2. Variables of Interest

The following information was collected on the outcome and independent variables. 

The outcome variable of interest was the ever presence/absence of bronchitis, based on the question: “Has a doctor ever said this child had bronchitis (yes/no)?” There are two kinds of bronchitis: acute and chronic. Both indicate inflammation of the bronchial tubes. Acute bronchitis lasts generally for a few weeks and often begins after a respiratory infection. Chronic bronchitis may last for years. In this analysis, any bronchitis acute or chronic was captured.

For the individual factors, the following demographic information was collected: child’s sex; child’s age; being the first born child (yes/no); breast feeding; birth weight; respiratory allergies, including allergies for house dust, grain dust, pollen, trees, grasses, mold or mildew, dogs, cats or bird feathers (yes/no); and whether or not the mother smoked during pregnancy. Any respiratory allergy was defined as a positive response to allergy to any of the following: house dust, grain dust, pollen, trees, grasses, mold or mildew, dogs, cats and bird feathers. Body weight was classified as not being overweight or obese, using the classifications of overweight and obese established by the International Obesity Task Force [23,24], and based on parental report of height and weight. 

For the contextual factors, information was collected on the following contextual factors: difficulty accessing regular or ongoing health care in the past 12 months (yes/no); exposure to parental smoking, either one or both parents (yes/no); any smoking in the home (yes/no); number of people living in the home; signs of mold or mildew in any living areas in home (yes/no); whether the house had any damage caused by dampness (e.g., wet spots on walls, floors) (yes/no); socioeconomic status based on the parents’ highest education level (<high school or ≥high school); housing conditions such as the presence of natural gas heating, air conditioner, air filter, humidifier in the home, dehumidifier in the home; and pet in the home. 

### 2.3. Statistical Analysis

Statistical analyses were conducted using SPSS version 24 (IBM SPSS Statistics for Windows, Armonk, NY, USA). Logistic regression models were used to predict the relationship between ever bronchitis (yes or no) and a set of explanatory variables. Based on the bivariable analysis, variables with *p* ≤ 0.20 became candidates for a multivariable model. The strength of associations was presented by adjusted odds ratios (OR_adj_) and their 95% confidence intervals (CI). After the fitting of the first multivariable model, variables that were statistically significant (*p* < 0.05) as well as important covariates were retained in the final multivariable model. Sex and age were not significant at the 20% level of significance. Since they are important biological variables, they were included in the multivariable analysis. A parsimonious model was selected based on the Hosmer–Lemeshow goodness-of-fit statistic [25]. 

## 3. Results

There were 351 First Nations children aged 6–17 years who participated in the FNLHP. The mean age and standard deviation of the study population was 10.7 ± 3.1 years. More girls (53.0%) than boys (47.0%) participated. The prevalence of ever bronchitis was 17.9% (63/351). Of those, 34.9% (22/63) had been hospitalized for breathing problems.

The univariate relationships between the environmental factors, individual factors or other covariates and ever bronchitis using unadjusted logistic regression are shown in Table 1. Obese children were at higher risk of ever having been diagnosed with ever bronchitis compared to children who were not overweight or obese. Children living in homes with signs of mold or mildew were also more likely to report bronchitis. Any respiratory allergy was a significant comorbid condition for ever bronchitis.

There was no statistically significant difference in prevalence of ever bronchitis by age, but there was a higher percentage (49.2%) of younger children (6 to 10 years) with reported ever bronchitis compared to older age groups (33.3% and 17.5%). A higher proportion of children (58/63, 92.1%) with bronchitis were exposed to parental tobacco smoking compared to children without ever bronchitis (85.4%). Body mass index, parental smoking (either one or both parents), parents’ highest education (either one or both parents), having an air conditioner and having a humidifier were selected using the *p* ≤ 0.2 rule and served as covariates for the multivariable model (Table 1).

Results of multivariable logistic regression analysis adjusted for covariates are presented in Table 2. The significant predictors of increased risk of ever bronchitis were being obese (OR = 3.90; 95% CI = 1.81–8.39), having respiratory allergies (2.29; 1.14–4.61), parental smoking (2.97; 1.03–8.54) and signs of mold or mildew in the home (2.02; 1.09–3.74) (Table 2). The Hosmer and Lemeshow test (χ^2^ = 11.77, degrees of freedom = 8, *p*-value = 0.162) indicates that numbers of children with ever bronchitis are not significantly different from those predicted by the model indicating a good model fit.

## 4. Discussion

Bronchitis is a common disease among children and adults and very few studies have been conducted with First Nations children [1,2,26]. In this study, we were able to assess the school aged children aged 6–17 years from two First Nations communities in Saskatchewan. We assumed that the question “Has a doctor ever said this child had bronchitis?” would capture all ever bronchitis cases (acute or chronic) from participant child’s infancy to the date of survey completion. Results showed a high prevalence of ever bronchitis (about 18%) among the study population. Most available reports are for chronic bronchitis. Therefore, we do not have enough information to compare any bronchitis (acute or chronic) with previous studies except one study by Senthilselvan et al. [2]. The authors reported that the prevalence of physician-diagnosed ever bronchitis was 15.6% in Canadian Aboriginal children aged 5 to 14 years and 13.7% in youth and youth adults aged 15 to 34 years in 1998. We observed that obesity, any respiratory allergy, parental cigarette smoking, and signs of mold or mildew in the home were significant risk factors for increased prevalence of bronchitis.

Housing quality is an important determinant of health status. The life span of reserve housing is short due to poor construction, lack of maintenance, and overcrowding in homes [27]. Physical living environments (housing quality, crowding in homes, residential dampness and mold, etc.) of First Nations people are different from those of other populations in Canada and are associated with adverse respiratory outcomes [28]. About 23.4% of First Nations adults live in overcrowded housing [29]. The First Nations RHS 2008–2010 reported that 37.5% of First Nations children were living in crowded homes [3]. According to the RHS 2008–2010, 37.3% of First Nations households required major repairs and 33.5% required minor repairs [3]. About 50.0% of First Nations adults reported having mold and mildew present in their homes [3,30]. Studies have shown that there is a correlation between dampness, increased damage, deterioration of the building and increased mold growth [31,32]. In our study, we observed high proportions of any damage caused by dampness (42.2%), signs of mold or mildew in the home (44.2%) and dampness in the home during the past 12 months (53%).

A review of the epidemiologic evidence by Mendell et al. [11] indicated a consistent association between dampness or mold and bronchitis. Fisk et al. [12] reported an association between residential dampness and mold with respiratory tract infections including bronchitis in children. A study from Western Germany indicated a 1.3 times higher risk (95% CI: 1.03–1.65) of ever being diagnosed with bronchitis in 6-year-old children exposed to damp housing conditions [33]. A positive association between bronchitis and residential dampness or mold with an OR of 1.38 (95% CI: 1.28–1.47) for children was reported by Antova et al. [10]. In 1991, a Canadian study conducted by Dales et al. indicated that homes with reported mold or dampness were associated with respiratory outcomes including bronchitis, with an OR of 1.32 (1.06–1.39) [34]. Similarly, this study reported that signs of mold or mildew in the home was a significant risk factor for increased prevalence of bronchitis in the group of First Nations children. Even though a positive association was consistent with other populations, we observed a higher risk (almost 2 times) of ever bronchitis among First Nations children living on reserve who reported signs of mold or mildew in the home compared to risks presented in the previous studies [10,33,34]. 

There was an association between report of allergy and bronchitis symptoms. There was a higher chance of respiratory infections including bronchitis among those suffering from severe allergies [35]. In our study, there was a higher risk of bronchitis in children who reported having any respiratory allergy. 

Some studies have shown that high body mass index was associated with bronchitis [36,37,38]. Lee et al. [36] reported an increased risk of occurrence of bronchitis among overweight and obese adolescents. In the current study, we observed a higher prevalence of obesity in children with ever bronchitis. A reduction in respiratory system compliance and an airflow limitation could be caused by excess body weight [39]. Obese individuals may take shallow breaths due to compression of the thoracic cage from high soft tissue weight and fatty infiltration of the chest wall, resulting in dyspnea, chronic cough or bronchitis [39].

Recent reports revealed that the smoking rate of First Nations individuals was 57% [40] compared with 14.6% among non-Aboriginal peoples [41], which was over 3 times higher. Aboriginal peoples were more likely than non-Aboriginal people to be exposed to environmental tobacco smoke in the home [42]. First Nations children were more likely to be exposed to environmental tobacco smoke compared to non-Aboriginal children [15]. Scientifically confirmed health risks to children from tobacco smoke include bronchitis, pneumonia and asthma [43]. Parents reported an increased prevalence of respiratory symptoms and an increased frequency of bronchitis and pneumonia early in life of their children if parents smoked cigarettes [43]. Several other authors also reported an increased risk of bronchitis in children with parents who smoke [44,45]. Our study reports a significant association between parental cigarette smoking and ever bronchitis, and 86.6% of children’s parents were currently smoking. 

The current study has several strengths and several limitations. It included large sample size of 351 children from two First Nations reserves in Saskatchewan. This study had moderate response rate (58.2%). In general, due to the cross-sectional nature of the study, one of the major limitations was the parent-reported survey recall bias of disease history. No detailed information on potential confounders of income status and day care attendance was available. Another key limitation of the study is that the dependent variable (bronchitis) is not well defined. Analysis is based on the question “has a doctor ever said this child had bronchitis?”. We do not have information to distinguish between the type of bronchitis as being acute, recurrent, or chronic. Therefore, the reported prevalence includes all types of bronchitis. Also, there is evidence that asthma diagnosis can be associated with a history of childhood bronchitis [46] and there can be considerable overlap with asthma and childhood bronchitis with respect to etiology and treatment.

We have carried out sensitivity analysis of bronchitis removing 25 cases with both diagnosis of ever asthma and bronchitis. Multivariable regression analysis results reveled that associations remained similar except for the association with any respiratory allergies which was no longer significant. This may be because cases with respiratory allergies were more strongly associated with asthma in those cases with asthma and bronchitis conditions. 

Another limitation is that objective measures of home inspections of child study participants are not available due to study budget limitations. Home assessments are currently being conducted on some homes in the community and results were not available for this analysis. Health literacy can be an issue when reporting the occurrence of specific chronic lung conditions. However, most of the parents completing the questions had an education level of high school or higher (88.3%). Also, the survey questionnaire was pre-tested with First Nations families not involved with the study and wording in the questionnaire was adjusted following the pre-test.

## 5. Conclusions

There are several modifiable risk factors that should be considered when examining preventive interventions for bronchitis including obesity, smoking exposure, and home mold or dampness. The high prevalence of damage caused by dampness and signs of mold or mildew in First Nations homes combined with a demonstrated relationship to bronchitis reveals a serious public health issue for First Nations communities.

## Figures and Tables

**Table 1:** Frequencies and unadjusted odds ratio (OR) for the relationship between risk factors and ever bronchitis.

Variable	Ever Bronchitis				
	Total (*n* = 351)	No (*n* = 288)	Yes (*n* = 63)	Unadjusted OR (95% Confidence Interval (CI)) *	*p*-Value
	*n* (%)	*n* (%)	*n* (%)		
Individual factors					
Sex					
Male	165 (47.0)	135 (46.9)	30 (47.6)	1.03 (0.60, 1.78)	0.915
Female	186 (53.0)	153 (53.1)	33 (52.4)	1.00	
Age group					
6–10 years	175 (49.9)	144 (50.0)	31 (49.2)	0.76 (0.35, 1.65)	0.703
11–14 years	126 (35.9)	105 (36.5)	21 (33.3)	0.71 (0.31, 1.60)	
≥15 years	50 (14.2)	39 (13.5)	11 (17.5)	1.00	
Body mass index					
Obese	71 (23.1)	50 (19.8)	21 (37.5)	**3.12 (1.57, 6.58)**	**0.005**
Overweight	90 (29.2)	72 (28.6)	18 (32.1)	1.91 (0.93, 3.94)	
Normal/underweight	147 (47.7)	130 (51.6)	17 (30.4)	1.00	
Birth weight					
Underweight	19 (7.1)	14 (6.4)	5 (10.0)	1.86 (0.63, 5.54)	0.204
Overweight	51 (19.0)	38 (17.4)	13 (26.0)	1.78 (0.86, 3.72)	
Normal	199 (74.0)	167 (76.3)	32 (64.0)	1.00	
Breast Fed					
Yes	185 (52.7)	148 (51.4)	37 (58.7)	1.35 (0.77, 2.34)	0.290
No	166 (47.3)	140 (48.6)	26 (41.3)	1.00	
First born					
Yes	93 (26.5)	80 (27.8)	13 (20.6)	0.68 (0.35, 1.31)	0.245
No	258 (73.5)	208 (72.2)	50 (79.4)	1.00	
Any respiratory allergies					
Yes	71 (20.2)	51 (17.7)	20 (31.7)	**2.16 (1.17, 3.98)**	**0.013**
No	280 (79.8)	237 (82.3)	43 (68.3)	1.00	
Difficulty of getting the regular or on-going health care					
Yes	17 (4.8)	11 (3.8)	6 (9.5)	2.65 (0.94, 7.46)	0.056
No	334 (95.2)	277 (96.2)	57 (90.5)	1.00	
Parents highest education (either one or both parents)					
<High school	41 (11.7)	37 (12.8)	4 (6.3)	0.46 (0.16, 1.34)	0.115
≥High school	310 (88.3)	251 (87.2)	59 (93.7)	1.00	
**Environmental factors**					
Smoke in home					
Yes	154 (43.9)	124 (43.1)	30 (47.6)	1.20 (0.70, 2.08)	0.509
No	47 (13.4)	164 (56.9)	33 (52.4)	1.00	
Mom smoke during pregnancy					
Yes	181 (51.6)	150 (52.1)	31 (49.2)	0.89 (0.52, 1.54)	0.679
No	170 (48.4)	138 (47.9)	32 (50.8)	1.00	
Parental smoking					
Yes	304 (86.6)	246 (85.4)	58 (92.1)	1.98 (0.75, 5.23)	0.168
No	47 (13.4)	42 (14.6)	5 (7.9)	1.00	
Number of people in home					
≤4	96 (27.9)	76 (27.0)	20 (32.3)	0.86 (0.47, 1.54)	0.399
>4	248 (72.1)	206 (73.0)	42 (67.7)	1.00	
Natural gas for heating					
Yes	249 (70.9)	206 (71.5)	43 (68.3)	0.86 (0.47, 1.54)	0.604
No	102 (29.1)	82 (28.5)	20 (31.7)	1.00	
Air conditioner					
Yes	113 (32.2)	87 (30.2)	26 (41.3)	1.62 (0.93, 2.84)	0.089
No	238 (67.8)	201 (69.8)	37 (58.7)	1.00	
Air Filter					
Yes	146 (41.6)	122 (42.4)	24 (38.1)	0.84 (0.48, 1.46)	0.534
No	205 (58.4)	166 (57.6)	39 (61.9)	1.00	
Humidifier					
Yes	49 (14.0)	36 (12.5)	13 (20.6)	1.82 (0.90, 3.68)	0.091
No	302 (86.0)	252 (87.5)	50 (79.4)	1.00	
Dehumidifier					
Yes	28 (8.0)	22 (7.6)	6 (9.5)	1.27 (0.49, 3.28)	0.617
No	323 (92.0)	266 (92.4)	57 (90.5)	1.00	
Wood fire place					
Yes	11 (3.1)	8 (2.8)	3 (4.8)	1.75 (0.45, 6.80)	0.413
No	340 (96.9)	280 (97.2)	60 (95.2)	1.00	
Pet in home					
Yes	164 (46.7)	133 (46.2)	31 (49.2)	1.13 (0.65, 1.95)	0.663
No	187 (53.3)	155 (53.8)	32 (50.8)	1.00	
Damage caused by dampness					
Yes	148 (42.2)	117 (40.6)	31 (49.2)	1.42 (0.82, 2.45)	0.212
No	203 (57.8)	171 (59.4)	32 (50.8)	1.00	
Signs of mold or mildew					
Yes	155 (44.2)	118 (41.0)	37 (58.7)	**2.05 (1.18, 3.57)**	**0.010**
No	196 (55.8)	170 (59.0)	26 (41.3)	1.00	

* Odds ratios that are significantly different from 1.00 (*p* < 0.05) are shown in bold.

**Table 2:** Adjusted odds ratio for the relationship between risk factors and ever bronchitis.

Variable	Ever Bronchitis	
	OR * (95% CI) ^†^	*p*-Value
Individual factors		
Body mass index		
Obese	**3.90 (1.81, 8.39)**	**0.001**
Overweight	2.01 (0.95, 4.27)	0.068
Normal/underweight	1.00	
Any respiratory allergies		
Yes	**2.29 (1.14, 4.61)**	**0.020**
No	1.00	
**Environmental factors**		
Parental smoking		
Yes	2.97 (1.03, 8.54)	0.043
No	1.00	
Signs of mold or mildew		
Yes	**2.02 (1.09, 3.74)**	**0.026**
No	1.00	

* Odds ratios that are significantly different from 1.00 (*p* < 0.05) are shown in bold. ^†^ Adjusted for age, sex and parents’ highest education.
